# Single-cell DNA methylation sequencing reveals epigenetic alterations in mouse oocytes superovulated with different dosages of gonadotropins

**DOI:** 10.1186/s13148-020-00866-w

**Published:** 2020-06-01

**Authors:** Ying Huo, Zhi Qiang Yan, Peng Yuan, Meng Qin, Ying Kuo, Rong Li, Li Ying Yan, Huai Liang Feng, Jie Qiao

**Affiliations:** 1 Center for Reproductive Medicine, Department of Obstetrics and Gynecology, Peking University Third Hospital, No. 49 North HuaYuan Road, Hai Dian District, Beijing, 100191 China; 2 Key Laboratory of Assisted Reproduction, Ministry of Education, No. 49 North HuaYuan Road, Hai Dian District, Beijing, 100191 China; 3 Beijing Key Laboratory of Reproductive Endocrinology and Assisted Reproduction, No. 49 North HuaYuan Road, Hai Dian District, Beijing, 100191 China; 4 Department of Physiology and Pathophysiology, School of Basic Medical Sciences, Peking University, No. 38 XueYuan Road, Haidian District, Beijing, 100191 China; 5 Peking-Tsinghua Center for Life Sciences, Peking University, Beijing, 100871 China; 6 National Clinical Research Center of Obstetrics and Gynecology, No. 49 North HuaYuan Road, Hai Dian District, Beijing, 100191 China; 7 The New York Fertility Center, New York Hospital Queens, Weill Medical College of Cornell University, New York, NY USA

**Keywords:** Single-cell DNA methylation sequencing, Epigenetic variations, Superovulation, Gonadotropins dosage

## Abstract

**Background:**

Epigenetic abnormalities caused by superovulation have recently attracted increasing attention. Superovulation with exogenous hormones may prevent oocytes from establishing an appropriate epigenetic state, and this effect may extend to the methylation programming in preimplantation embryos, as de novo DNA methylation is a function of developmental stage of follicles and oocyte size. Follicle-stimulating hormone (FSH) and human menopausal gonadotropin (hMG) are common gonadotropins used for superovulation, and appropriate concentrations of these gonadotropins might be necessary. However, no systematic study on the effects of DNA methylation alterations in oocytes associated with superovulation with different dosages of FSH/hMG at the single-cell level has yet been reported. In the current study, different dosages of FSH/hMG combined with human chorionic gonadotropin (hCG) were used in female mice to generate experimental groups, while naturally matured oocytes and oocytes superovulated with only hCG were respectively used as controls. Single-cell level DNA methylation sequencing was carried out on all these matured oocytes.

**Results:**

In this study, we revealed that the genome-wide methylation pattern and CG methylation level of the maternal imprinting control regions of all mature oocytes were globally conserved and stable. However, methylation alterations associated with superovulation were found at a specific set of loci, and the differentially methylated regions (DMRs) mainly occurred in regions other than promoters. Furthermore, some of the annotated genes in the DMRs were involved in biological processes such as glucose metabolism, nervous system development, cell cycle, cell proliferation, and embryo implantation and were altered in all dosages of FSH/hMG group (for example, *Gfod2* and *SYF2*). Other genes were impaired only after high gonadotropin dosages (for instance, *Sox17* and *Phactr4*).

**Conclusions:**

In conclusion, the current study addressed the effects of superovulation on DNA methylation from the perspective of different dosages of gonadotropins at the single-cell level. We found that the genome-wide DNA methylation landscape was globally preserved irrespective of superovulation or of the kind and dosage of gonadotropins used, whereas the methylation alterations associated with superovulation occurred at a specific set of loci. These observed effects reflect that superovulation recruits oocytes that would not normally be ovulated or that have not undergone complete epigenetic maturation. Our results provide an important reference for the safety assessment of superovulation with different dosages of gonadotropins. However, it should be noted that this study has some limitations, as the sample number and library coverage of analyzed oocytes were relatively low. Future studies with larger sample sizes and high-coverage libraries that examine the effects of superovulation on embryo development and offspring health as well as the underlying mechanisms are still needed.

## Background

Assisted reproductive technologies (ARTs) have been increasingly used to overcome fertility problems since the first birth in 1978 [1]. Although most newborns derived from ARTs seem healthy, substantial studies have reported that increased incidences of birth defects [2], congenital anomalies [3], impaired glucose metabolism [4], insulin resistance [5], cardiovascular diseases [4, 6], genetic abnormalities [7], epigenetic anomalies [8–10], and imprinting disorders such as Beckwith-Wiedemann syndrome (BWS), Silver-Russell syndrome (SRS), Angelman syndrome (AS), and Prader-Willi syndrome (PWS) [11, 12] are associated with ART procedures.

Superovulation with exogenous gonadotropins to induce follicle maturation and stimulate oocyte growth is the first procedure and the most important phase of an ART cycle [13]. Increasing evidence has revealed that superovulation alters DNA methylation [14, 15], increases chromosomal aneuploidy [16], perturbs genomic imprinting [17], affects endometrial receptivity [18], decreases implantation rate [19], delays embryo development [9, 20], and impairs offspring health [21, 22].

In particular, DNA methylation is an epigenetic marker that can be established de novo, maintained through cell division and interpreted by transcription machinery and DNA-binding proteins, playing a critical role in the process of gametogenesis and embryo development [23]. In mice, de novo DNA methylation is a function of developmental stage of follicles and oocyte size; it begins in oocytes at around 10 days after birth or when an oocyte reaches at least 40–45 μm in diameter and becomes established at approximately 21 days of age, at the germinal vesicle (GV) stage, mainly via the de novo DNA methyltransferases DNMT3A and DNMT3L [14, 23–25]. Therefore, there is a strong possibility that superovulation, which mainly promotes oocyte maturation with exogenous hormones, may prevent oocytes from establishing an appropriate epigenetic state, as Saenz-de-Juano et al. showed in a representative study in mice [14].

Furthermore, the oocyte methylome is unique in that it predominantly includes genic regions, with almost no methylation in intergenic regions [23]. As the most important genic regions for methylation, CpG islands (CGIs) not only constitute the germline differentially methylated regions (gDMRs) of imprinted genes that retain monoallelic methylation in a parent-of-origin specific manner following fertilization [14, 26–28] but also regulate the trophoblast lineage in mice [29] and placental-specific imprinting in humans [30]. Evidence has been increasing that superovulation may contribute to epigenetic changes not only in oocytes but also in developing embryos [31]. Moreover, Saenz-de-Juano et al. [32] indicated that the loss of imprinted DNA methylation in mouse blastocysts was inflicted to a similar extent by superovulation. Therefore, the effects of superovulation on the epigenetic programming of an oocyte during its growth within a follicle would extend to methylation programming in preimplantation embryos, which is associated with offspring health.

Follicle-stimulating hormone (FSH), human menopausal gonadotropin (hMG), human chorionic gonadotropin (hCG), and luteinizing hormone (LH) are exogenous gonadotropins commonly used in clinical superovulation. They play crucial roles in regulating folliculogenesis and follicle maturation [33–35]. FSH is derived from either urinary sources or recombinant (rFSH) techniques and hCG is largely produced in the placenta during pregnancy whereas hMG, which has both LH and FSH activity, is extracted from postmenopausal urine [35, 36]. Until now, the effects of different gonadotropins have remained controversial. Hompes et al. [37] showed that hMG resulted in a lower incidence of hyper-response. Furthermore, Coomarasamy et al. [38] presented a pooled 4% increase in live birth rate after the use of by hMG relative to rFSH, whereas Ararooti et al. [39] reported that the FSH protocol provided a better superovulatory response and a higher number of embryos. Until now, there has been no systematic study of the similarities and differences in DNA methylation alterations associated with superovulation using FSH/hMG + hCG.

Furthermore, an appropriate gonadotropin concentration is necessary for oocyte developmental competence and resulting embryo quality. A recent report suggested that the difference between recombinant and urinary-derived highly purified hMG/FSH in the required amount to reach a live birth appeared small [13]. However, high-concentration gonadotropin might significantly increase first meiotic division errors and result in more aneuploidy in oocytes [16]. Our previous study also showed that high FSH impaired oocyte maturation competence, spindle assembly, blastocyst formation and implantation as well as viable pup production, although the physiological indices and behaviors of the offspring seemed not to be influenced [40]. However, the effects of high-concentration gonadotropin on genome-wide methylation programming remain unknown.

The present study aimed to investigate the effects of superovulation with different concentrations of gonadotropin on genome-wide DNA methylation during oocyte maturation. Thus, different dosages of FSH/hMG combined with hCG were used in female adult mice to obtain metaphase II (MII) oocytes, while naturally ovulated oocytes and oocytes superovulated with only hCG were used as controls. Single-cell DNA methylation sequencing was carried out to characterize the DNA methylation pattern in each group.

## Results

### The whole-genome DNA methylation pattern was unaffected by superovulation

**Fig. 1:**
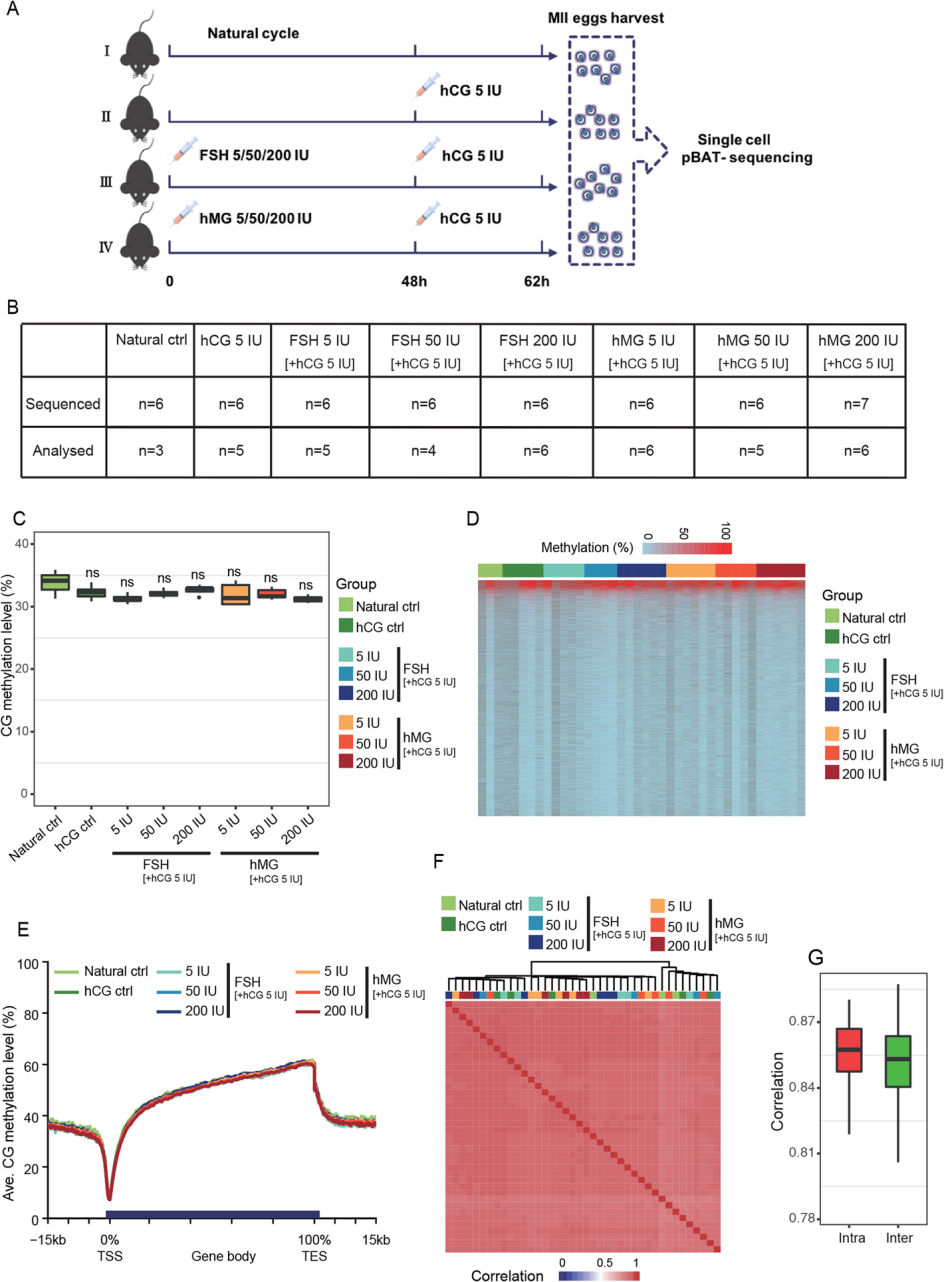
Analysis of whole-genome DNA methylation profiles. **a** Experimental design of the current study. I, Natural control: natural MII oocytes were harvested without any hormone treatment. II, hCG control: mice were treated with 5 IU hCG, and then the MII oocytes were harvested. III, FSH group: mice were intraperitoneally injected with 5/50/200 IU FSH. Forty-eight hours later, 5 IU hCG was injected, and after 14 h, the MII oocytes were harvested. IV, hMG group: mice were intraperitoneally injected with 5/50/200 IU hMG. Forty-eight hours later, 5 IU hCG was injected, and after 14 h, the MII oocytes were harvested. The methylome of each single oocyte was quantified with single-cell pBAT sequencing. **b** Sample sizes of the groups used in the current study. **c** Boxplot of whole-genome CG methylation levels. ns: not significantly different compared with natural control. **d** Heatmap of average promoter methylation levels. Each row represents a gene promoter and each column represents an oocyte. The gonadotropin treatment for each oocyte is shown at the top of the heatmap. **e** Average DNA methylation levels of gene bodies. The genomic compartments of the oocytes included 15 kb upstream of the transcription start sites (TSS) and 15 kb downstream of the transcription end sites (TES). **f** Pearson correlation clustering of the oocytes. The gonadotropin treatment of each oocyte is shown on the top of the heatmap, while the cluster tree shows methylome similarity. **g** Boxplot showing the correlations within (intra) and between (inter) the oocyte groups with different gonadotropin treatments

To investigate the potential effects of different dosages of FSH/hMG combined with hCG on methylation patterns during oocyte maturation, we carried out single-cell whole-genome DNA methylation sequencing on individual oocytes. The experimental design is summarized in Fig. 1a. For each oocyte, we obtained 9.11 Gb sequencing data on average, covering 4.59 million CG sites (≥ 1×). For the following analysis, we excluded oocytes with less than 1 million covered CG sites or less than 10% mapping efficiency; the sample sizes of each group are presented in Fig. 1b. The quality of the data including mapping rate, bisulfite conversion rate, and covered CG site number is summarized in Additional file 1: Figure S1, along with histograms of the distribution of CG methylation values.

The whole-genome CG methylation levels of the oocytes showed no significant difference (*p* value >0.05, ANOVA), although the whole-genome GG methylation levels of the mature oocytes derived from superovulation seemed slightly lower than those of the naturally ovulated oocytes (Fig. 1c). Additionally, we did not detect an obvious change (*p* value > 0.05, ANOVA) in CHG methylation level (Additional file 2: Figure S2A) or CHH methylation level (Additional file 2: Figure S2B). In agreement with the global analysis, the heatmap visualization of CG methylation status revealed that the promoters of the oocytes from each group did not display any clear alterations (Fig. 1d), nor did the genomic compartments including 15 kb upstream of the transcription start sites (TSS) and 15 kb downstream of the transcription end sites (TES) (Fig. 1e). However, it is worth noting that the global CpG methylation rate of the naturally ovulated oocytes appeared to be higher than those of all the other groups, although no statistical significance was found due to the small sample size (Fig. 1c). Additionally, an analysis of the global DNA methylation pattern using Pearson correlation clustering indicated that there was no clear segregation associated with superovulation (Fig. 1f).

Furthermore, we also analyzed the CG methylation levels of oocyte-methylated CpG island (metCGI), unmethylated CpG island (unmetCGI), promoter, exon, intron, intragenic region, intergenic region, LINE, SINE, LTR, rRNA, and tRNA. Again, we observed no significant difference among the groups (Additional file 3: Figure S3A). Moreover, we found that the CG methylation levels of some maternal imprinting control regions (ICRs), including *Igf2R*, *U2afq-rs1*, *Gnas*, *Impact*, *Snrpn*, *Peg3*, *Grb10*, *Nespas-Gnasxl*, *Peg10*, *Mest*, *Kcnq10t1*, and *Zac1* did not show obvious differences (Additional file 3: Figure S3B). Likewise, the chromosome copy number (Additional file 4: Figure S4) did not differ across all these groups.

However, the correlation analysis of the oocytes revealed that the biological replicates within the groups showed relatively higher correlation (Fig. 1g). The results suggested that there was a degree of methylation difference between the experimental groups.

### Alterations in the DNA methylation pattern caused by FSH treatment

**Fig. 2:**
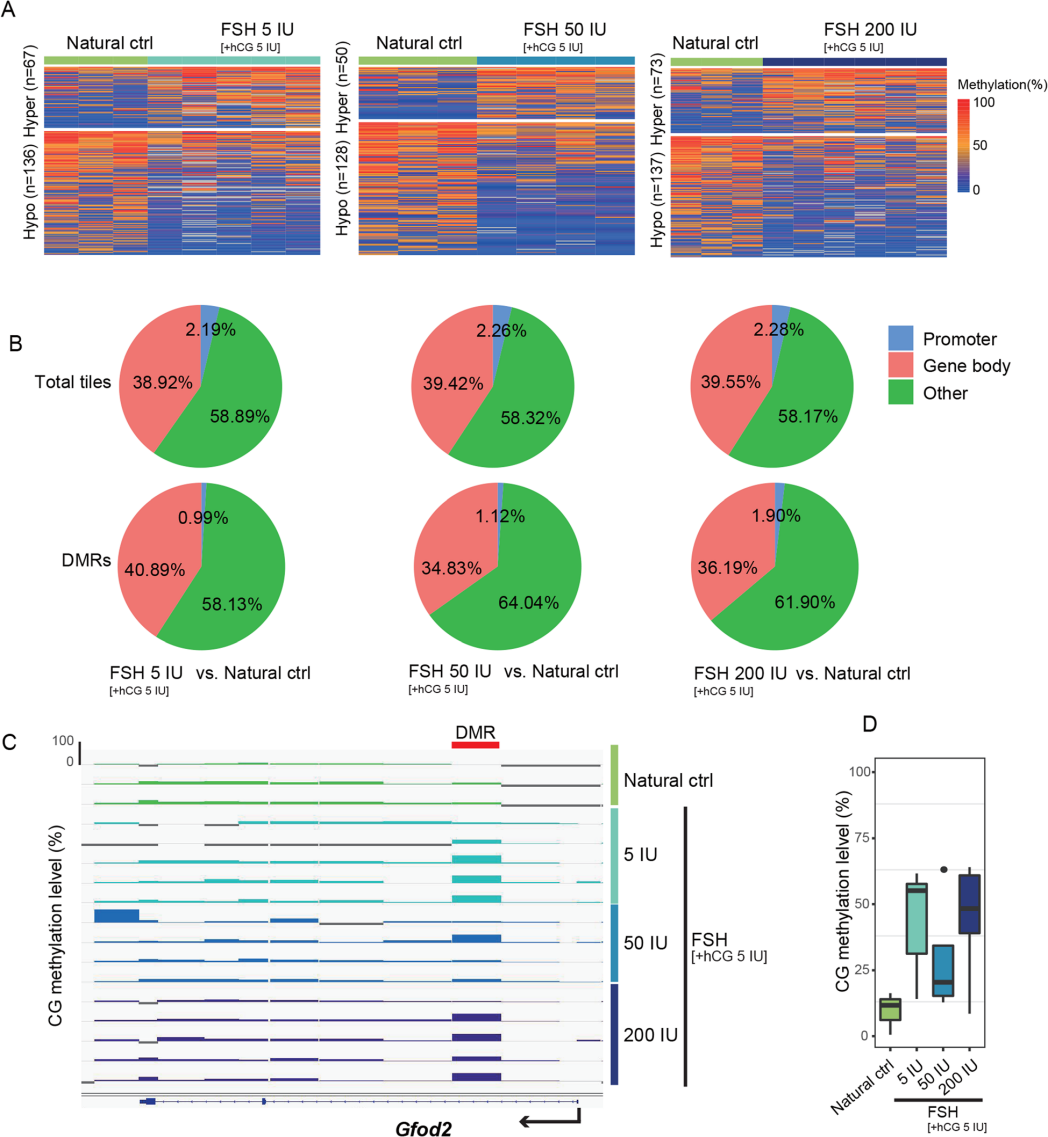
Alterations in DNA methylation after superovulation with different dosages of FSH (vs. natural control). **a** Heatmap of differentially methylated regions (DMRs) (100-CpG window size, corrected *p* value < 0.05, difference > 20%) between each FSH group and natural control group. Left panel, FSH 5 vs. natural control. Middle panel, FSH 50 vs. natural control. Right panel, FSH 200 vs. natural control. **b** Pie chart showing the genomic distribution of informative tiles (top) and DMRs (bottom) in each pairwise comparison. **c** Integrative Genomic Viewer (IGV) screenshot of a 68 kb region showing methylation at the *Gfod2* locus, with one tile consistently hypermethylated in the FSH 5 IU, FSH 50 IU, and FSH 200 IU groups. Each vertical bar in the screenshot represents the methylation value (range, 0–100%) of a non-overlapping 100-CpG tile. Genes are shown at the bottom of the screenshot. The treatment of each oocyte is shown on the right of the screenshot. **d** Boxplot of the methylation levels of the consistently hypermethylated DMRs shown in **c**

To investigate the effects of FSH on the oocyte methylome, we identified differentially methylated regions (DMRs) with 100-CpG tiling in each FSH dosage group. To reduce false discovery of DMRs, we performed comparisons between random combinations of the samples. The relationship between the number of DMRs identified by methylKit and the corrected *p* value is presented in Additional file 5: Figure S5. As shown in Additional file 5: Figure S5, the number of DMRs in the randomized group was comparable to that in the real group. Therefore, we focused on the recurrent DMRs in each treatment group. Compared with the natural control group, 67 hyper- and 136 hypo-DMRs in the FSH 5 IU group, 50 hyper- and 128 hypo-DMRs in the FSH 50 IU group, and 73 hyper- and 137 hypo-DMRs in the FSH 200 IU group were found (Fig. 2a). We further investigated the genomic distribution of informative tiles and DMRs relative to the natural control. The percentages of informative tiles among all the tiles were as follows: in the FSH 5 IU group, 2.19% in promoters, 38.92% in gene bodies, and 58.89% in other regions; in the FSH 50 IU group, 2.26% in promoters, 39.42% in gene bodies, and 58.32% in other regions; and in the FSH 200 IU group, 2.28% in promoters, 39.55% in gene bodies, and 58.17% in other regions (Fig. 2b). The percentages of DMRs were as follows: in the FSH 5 IU group, 0.99% in promoters, 40.89% in gene bodies, and 58.13% in other regions; in the FSH 50 IU group, 1.12% in promoters, 34.83% in gene bodies, and 64.04% in other regions; and in the FSH 200 IU group, 1.90% in promoters, 36.19% in gene bodies, and 61.90% in other regions (Fig. 2b). These results showed that DMRs are not enriched in promoters, indicating that the majority of DMRs did not disturb the core regions related to gene transcription.

The genes annotated in the hyper-/hypo-DMRs are listed in Additional file 6: Supplementary Table S1. Certain genes, such as *Gfod2*, *Foxi3*, *Celf4*, and *SYF2*, which are involved in some important biological processes, such as glucose metabolism, nervous system development, cell cycle, cell proliferation, and mRNA processing, were found to be altered in all the FSH groups relative to the natural control group (Additional file 6: Supplementary Table S1). Notably, an Integrative Genomic Viewer (IGV) screenshot of a 68 kb region showed that the *Gfod2* locus, which encodes a glucose-fructose oxidoreductase, was hypermethylated in all the FSH groups, although the FSH 50 IU group presented relatively low methylation levels compared with the FSH 5 IU and 200 IU groups (Fig. 2c and d). The CpG distribution of the *Gfod2* DMR in the FSH groups is shown in Additional file 7: Figure S6.

The corresponding alterations in DNA methylation after superovulation with different dosages of FSH compared with the hCG control group are presented in Additional file 8: Figure S7. Notably, *Gfod2* was also hypermethylated in all the superovulated oocytes relative to the hCG control oocytes, while the FSH 50 IU group presented relatively low methylation levels compared with the FSH 5 IU and 200 IU groups (Additional file 8: Figure S7C and S7D).

Furthermore, we analyzed methylation variability across the controls and FSH groups. We calculated the standard deviation of methylation within the biological replicates for 10,000 randomly selected 100-CpG tiles. The tiles in the natural control group, hCG control group, FSH 5 IU group, FSH 50 IU group, and FSH 200 IU group had a range of only 0.06–0.08 (median) standard deviations (Additional file 9: Figure S8A). Additionally, the cumulative distribution curve showed a similar distribution of standard deviations among these groups (Additional file 9: Figure S8B). To explore whether specific regions of the genome were affected by FSH superovulation, we also evaluated the standard deviations of the methylation levels in high-, medium-, and low-methylation CGIs in oocytes of each group. As shown in Figure S8C (Additional file 9), the standard deviations of the high- and low-methylation CGIs were relatively small, whereas the variances of the medium-methylation CGIs were relatively large. However, this effect on the standard deviation seemed stable, and there was no significant change with FSH dosage (Additional file 9: Figure S8C). In addition, we calculated the distribution of distance between the top 10,000 variable 100-CpG tiles and the nearest TSS. We found that most tiles in each group had a > 10K distance from the nearest TSS, indicating that the core regions related to gene transcription were unlikely to be disturbed (Additional file 9: Figure S8D).

### Alterations in the DNA methylation pattern caused by hMG treatment

**Fig. 3:**
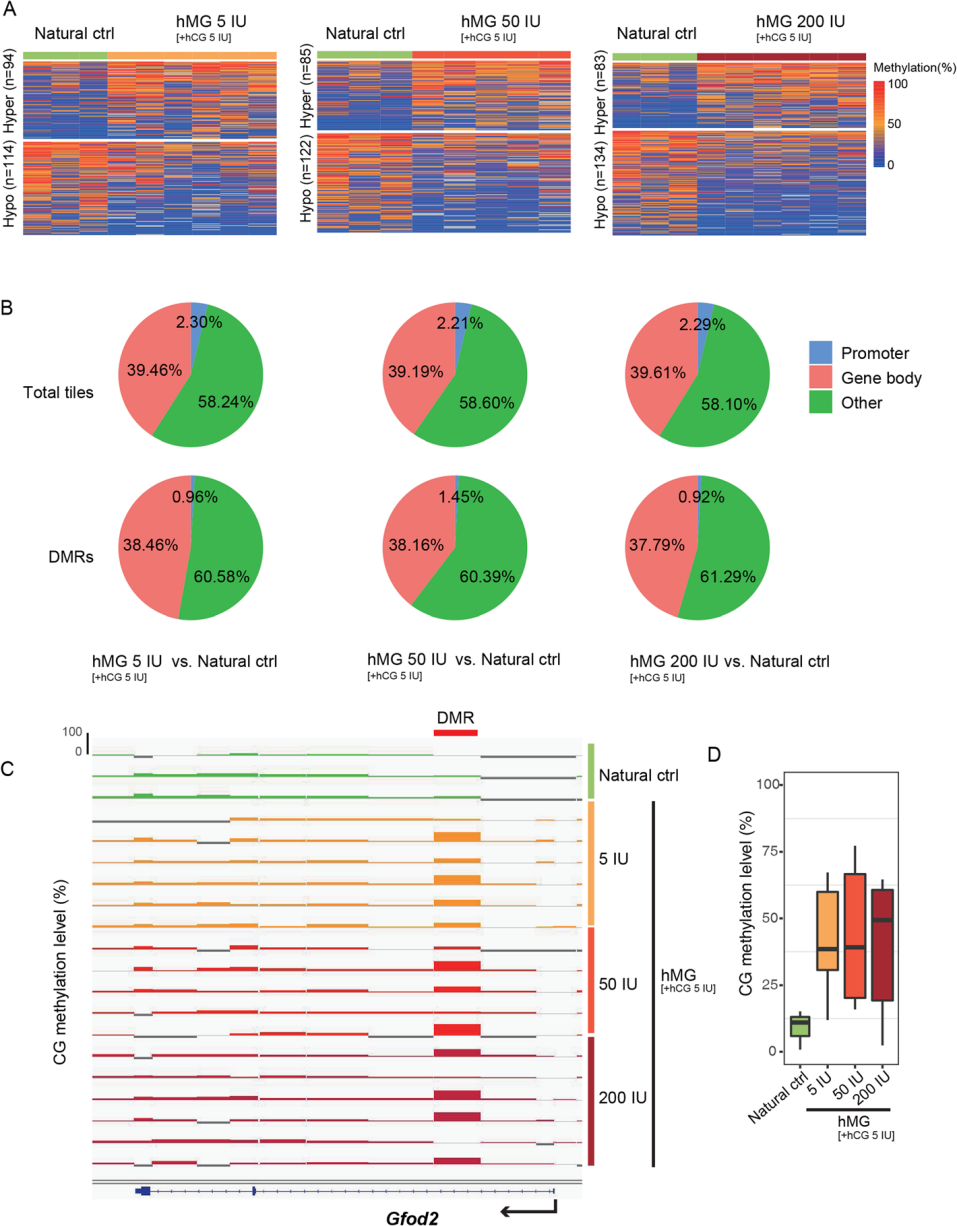
Alterations in DNA methylation after superovulation with different dosages of hMG (vs. natural control). **a** Heatmap of differentially methylated regions (DMRs) (100-CpG window size, corrected *p* value < 0.05, difference > 20%) between each hMG group and natural control group. Left panel, hMG 5 vs. natural control. Middle panel, hMG 50 vs. natural control. Right panel, hMG 200 vs. natural control. **b** Pie chart showing the genomic distribution of informative tiles (top) and DMRs (bottom) in each pairwise comparison. **c** Integrative Genomic Viewer (IGV) screenshot of a 68 kb region showing methylation at the *Gfod2* locus, with one tile consistently hypermethylated in the hMG 5 IU, hMG 50 IU, and hMG 200 IU groups. Each vertical bar in the screenshot represents the methylation value (range, 0–100%) of a non-overlapping 100-CpG tile. Genes are shown at the bottom of the screenshot. The treatment of each oocyte is shown on the right of the screenshot. **d** Boxplot of the methylation levels of the consistently hypermethylated DMRs shown in **c**

Similarly, we identified the DMRs with 100-CpG tiles in each hMG dosage group. Compared with the natural control group, 94 hyper- and 114 hypo-DMRs in the hMG 5 IU group, 85 hyper- and 122 hypo-DMRs in the hMG 50 IU group, and 83 hyper- and 134 hypo-DMRs in the hMG 200 IU group were found (Fig. 3a). We also investigated the genomic distributions of informative tiles and DMRs in each hMG group relative to the natural control. Similar to the FSH treatment, the percentages of total tiles were as follows: in the hMG 5 IU group, 2.30% in promoters, 39.46% in gene bodies, and 58.24% in other regions; in the hMG 50 IU group, 2.21% in promoters, 39.19% in gene bodies, and 58.60% in other regions; and in the hMG 200 IU group, 2.29% in promoters, 39.61% in gene bodies, and 58.10% in other regions (Fig. 3b). The percentages of DMRs were as follows: in the hMG 5 IU group, 0.96% in promoters, 38.46% in gene bodies, and 60.58% in other regions; in the hMG 50 IU group, 1.45% in promoters, 38.16% in gene bodies, and 60.39% in other regions; and in the hMG 200 IU group, 0.92% in promoters, 37.79% in gene bodies, and 61.29% in other regions (Fig. 3b).

The genes annotated in the hyper-/hypo-DMRs are listed in Additional file 10: Supplementary Table S2. Similar to the results of FSH treatment, certain genes involved in important biological processes, such as glucose metabolism, nervous system development, cell cycle, and cell proliferation were found to be changed in all the hMG groups relative to the natural control group (Additional file 10: Supplementary Table S2). In particular, *Gfod2*, which was hypermethylated in the FSH 5 IU and 200 IU groups, was also hypermethylated in all the hMG groups (Fig. 3c), and the relative methylation levels are shown in Fig. 3d. The CpG distribution in the *Gfod2* DMR in the hMG groups is shown in Additional file 11: Figure S9.

Correspondingly, the comparisons of DNA methylation among the different hMG dosage groups and the hCG control group are shown in Additional file 12: Figure S10. Notably, *Gfod2* was also hypermethylated in all the hMG groups relative to the hCG control (Additional file 12: Figure S10C ,D).

Again, we analyzed the variability in methylation across the control and hMG groups. Ten thousand randomly selected 100-CpG tiles from the natural control, hCG control, hMG 5 IU, hMG 50 IU, and hMG 200 IU groups still had a range of only 0.06–0.08 (median) standard deviations (Additional file 13: Figure S11A). The cumulative distribution curve also showed a similar distribution of standard deviations across each group (Additional file 13: Figure S11B). In accordance with the results of FSH superovulation, the standard deviations of high-, medium-, and low-methylation CGIs in oocytes were stable, and there was no significant change between the hMG groups, although the variance of the medium-methylation CGIs seemed relatively large (Additional file 13: Figure S11C). The core regions related to gene transcription were not impaired, and the most variable tiles in each group had a > 10K distance from the nearest TSS (Additional file 13: Figure S11D).

### Comparison of the effects of FSH and hMG superovulation on DNA methylation patterns

**Fig. 4:**
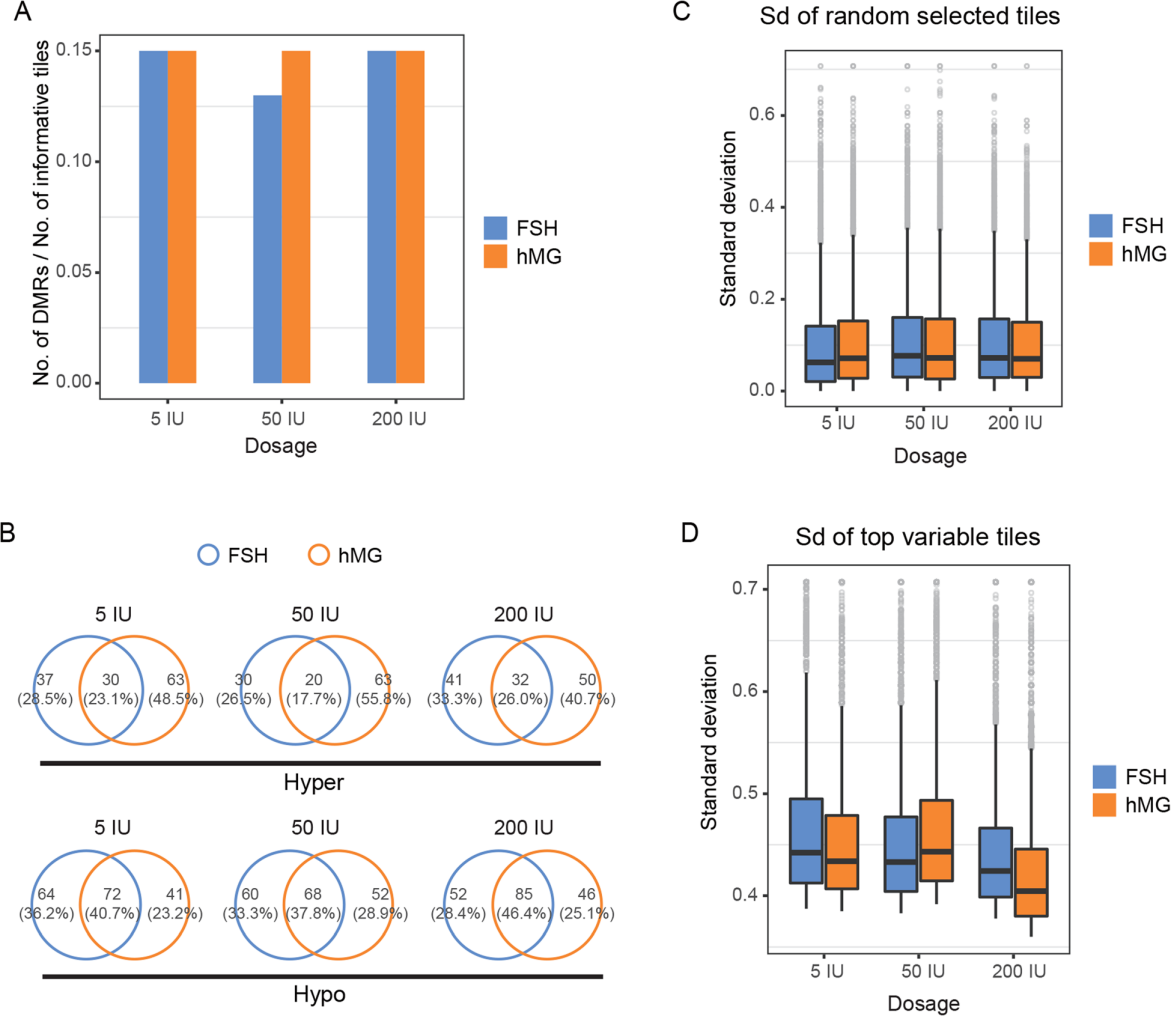
Comparison of DNA methylation patterns between FSH and hMG superovulation (vs. natural control). **a** Ratio of the identified DMRs and the covered informative regions of each FSH and hMG group. **b** Venn diagram of the overlap between genes annotated in hyper-/hypo-DMR in the FSH and hMG groups with the same dosage. **c** Standard deviation of the methylation level of 10,000 randomly selected 100-CpG tiles. **d** Standard deviation of the methylation level of the top 10,000 100-CpG tiles

The ratio of the identified DMRs and the covered informative regions of each FSH and hMG group relative to the natural control are presented in Fig. 4a. The consistency of the ratio indicated that there was no obvious increase in the number of DMRs associated with superovulation. The overlap ratio of the genes annotated in the hyper-/hypo-DMRs between the FSH and hMG groups with the same dosage is presented in Fig. 4b, and these genes are listed in Supplementary Table S3 (Additional file 14).

Remarkably, 34 genes annotated in the hyper-/hypo-DMRs, including *Gfod2*, *Adarb2*, *Agpat4*, *Alpk3*, *Arhgef15*, *Bean1*, *Celf4*, *Chd1*, *Crispld2*, *Dab1*, *Dctn6*, *Dusp27*, *Fam196a*, *Fat1*, *Fbln7*, *Foxi3*, *Gfod2*, *Gm4814*, *Gm5544*, *Gm5907*, *Gm833*, *Gng4*, *Lrtm2*, *Mir3963*, *Nup133*, *Paqr9*, *Phactr2*, *Rbm14*, *Runx1*, *Sipa1l2*, *SYF2*, *Tbx5*, *Tmem45a2*, *Unc5d*, and *Zfp507*, and some noncoding RNAs were found in all the FSH and hMG groups (vs. natural control), suggesting that these genes may be susceptible to superovulation.

Moreover, we also found that 23 other important genes, including *Ajap1*, *Aldh8a1*, *Amigo2*, *Cox6c*, *Gm38404*, *Kbtbd8*, *Kif3c*, *Krtap4-7*, *Lyzl4os*, *Mcc*, *Melk*, *Mir124a-2*, *Myrip*, *Phactr4*, *Plxnc1*, *Prr30*, *Rnf150*, *Shroom3*, *Sox17*, *Suds3*, *Trmt10b*, *Wdr41*, and *Zfp326*, and some noncoding RNAs were impaired in only the high dosage groups (50/200 IU vs. natural control), suggesting that they might represent a molecular marker for the corresponding impairments associated with high gonadotropin dosages.

Additionally, we compared the standard deviation of the methylation level between FSH and hMG treatments with the same dosage relative to the natural control and found that the standard deviations of 10,000 randomly selected 100-CpG tiles and the top 10,000 100-CpG tiles presented no obvious alterations (Fig. 4c and d).

The genes annotated in common in the hyper-/hypo-DMRs of the FSH and hMG groups with the same dosage relative to the hCG control group are listed in Additional file 14: Supplementary Table S3.

## Discussion

One important study of the effects of superovulation on genome-wide DNA methylation in oocytes was recently conducted by Saenz-de-Juano et al. [14]; it indicated that oocytes from superovulated adult females differed very little from naturally ovulated oocytes, although regions other than imprinted gDMRs were susceptible to methylation alterations associated with superovulation. In the current study, we showed that the genome-wide methylation patterns and the CG methylation levels of maternal ICRs in all the mature oocytes obtained naturally or from superovulation were globally conserved at the single-cell level and comprised alternating hyper- and hypomethylated domains. These results confirmed and supported those of previous studies.

However, a correlation analysis within (intra) and between (inter) all the oocyte groups revealed that the biological replicates clustered together within each group, whereas different gonadotropin treatments clustered differently, suggesting consistency across the variations in DNA methylation derived from different means of superovulation.

We detected specific differences in the DNA methylation of oocytes derived from superovulation relative to naturally matured oocytes. In mice, de novo DNA methylation in oocytes occurs progressively from the second follicle stage, while methylation is increased in the latter phase of growth, especially in transcriptionally active gene bodies [14, 24]. The results of the current study show that superovulation recruits oocytes that would not normally be ovulated or that have not undergone complete epigenetic maturation. The possible mechanistic origin of the DMRs, especially the hypomethylated DMRs, might be attributed to incomplete DNA methylation acquisition, as superovulation with exogenous hormones may prevent oocytes from establishing an appropriate epigenetic state and tend to recruit oocytes that have not fully completed the process of de novo methylation.

Furthermore, some of the genes annotated in the DMRs were involved in the regulation of biological processes, such as glucose metabolism, nervous system development, cell cycle, cell proliferation, and mRNA processing, and these were found to be disturbed at all dosages of FSH/hMG. In particular, *Gfod2*, glucose-fructose oxidoreductase domain containing 2, was hypermethylated in all superovulated oocytes. Variation in *Gfod2* has been suggested to contribute to the genetic basis for a differential response to a cholesterol- or lipid-lowering diet [41]. Currently, increasing evidence suggests that ARTs are associated with metabolic dysfunction. Ceelen et al. [4] reported higher fasting glucose levels, which were associated with an increased risk of metabolic dysfunction in postnatal life, in pubertal IVF-conceived children than in naturally conceived children. Wang et al. [42] provided evidence that superovulation increased lipid accumulation and decreased fatty acid synthesis. Furthermore, our unpublished studies also indicated that ARTs might have transgenerational effects on glucose metabolism and insulin resistance in mice. We speculate that *Gfod2* might be an important gene underlying the association between ARTs and metabolic dysfunction in the resulting offspring. Additionally, *Foxi3*, a member of the Forkhead family, was hypomethylated in all the superovulated oocytes and is known to affect embryogenesis and development [43]. CUGBP, ELAV-like family member 4 (*Celf4*), another gene that was hypomethylated in all the FSH/hMG groups, has also been indicated to play an important role in regulating and shaping the activity of the nervous system [44]. Moreover, *SYF2*, which was hypomethylated in all superovulated oocytes, has been reported to be involved in cell cycle progression and in pre-mRNA splicing [45], while disruption of the murine mp29/Syf2/ntc31 gene would result in embryonic lethality with an aberrant checkpoint response [46].

Our previous study using a mouse model showed that high FSH impaired oocyte maturation competence, spindle assembly, blastocyst formation and implantation as well as viable pup production, although the physiological indices and behaviors of the offspring seemed not to be influenced [13]. Xu et al. [16] claimed that high-concentration gonadotropin might significantly increase aneuploidy in human oocytes. Wang et al. [42] also indicated that superovulation affected lipid and fatty acid metabolism in a dose-dependent manner. Our current study indicated that the DNA methylation of specific genes in mature oocytes derived from high-concentration FSH/hMG + hCG was disturbed whereas the genome-wide methylation landscape seemed to be preserved irrespective of high gonadotropin dosages. These results confirmed and further expanded those of the previous studies from the perspective of DNA methylation.

Moreover, we found that some of the genes that were impaired only after a high dosage of gonadotropins played an important role in important biological processes such as embryonic stem cell differentiation, embryo implantation, cell proliferation, and nervous system development. For example, SRY-related HMG box gene 17 (*Sox17*) is a key player in human endometrial receptivity, mouse embryonic stem cell differentiation, and embryo implantation [47, 48]*.* In the current study, hypomethylated *Sox17* was found in all the high dosage groups. In addition, the Kruppel-associated box-zinc finger protein (KRAB-ZFP) family, the largest class of transcriptional regulators in the mouse, contributes to the early embryonic establishment of site-specific DNA methylation patterns [49] and multiple indicators related to female reproduction [50]. In our study, the methylation of *Zfp326*, an important member of the KRAB-ZFP family, was altered in the high-dosage FSH+ hCG/hMG + hCG groups, while existing evidence has revealed that *Zfp326* promotes cancer cell proliferation [51]. Moreover, Shroom family member 3 (*Shroom3*) and phosphatase and actin regulator 4 (*Phactr4*), which are involved in the regulation of neural tube closure, were also hypomethylated in all the high-dosage groups. *Shroom3* has been found to regulate myosin II distribution and cellular organization during neural tube closure [52], while *Phactr4* regulates neural tube and optic fissure closure through differential regulation of PP1 and the cell cycle [53]. Therefore, the existing evidence strongly argues that superovulation, especially with a high dosage of gonadotropins, would impair oocyte maturation and, thus, preimplantation embryo development, possibly by disrupting the epigenetic patterns of related regulatory genes.

However, this study has some limitations, as the number and library coverage of the analyzed oocytes were relatively low. Future studies with larger sample sizes and higher library coverage are still needed.

## Conclusions

In conclusion, the current study discussed the safety of superovulation from the perspective of DNA methylation and at the single-cell level. We further confirmed that the genome-wide DNA methylation landscape was globally preserved irrespective of superovulation as well as the kind and dosage of gonadotropins used. Importantly, we did detect that the methylation alterations associated with superovulation occurred at a specific set of loci and that gene body regions seemed more susceptible than promoters. Furthermore, the DNA methylation differences in some important genes, especially those impaired only after a high dosage of gonadotropins, that were involved in important biological processes such as glucose metabolism, nervous system development, cell cycle, cell proliferation, and embryo implantation, might reflect the transcriptional immaturity of the oocytes recruited by superovulation. However, due to the limitations of this study with respect to low sample number and library coverage, future studies with larger sample sizes and higher coverage libraries are still needed to verify the current findings and explore the effects of superovulation on embryo development and offspring health as well as the underlying mechanisms.

## Methods

### Animals

Female C57BL/6 mice at an age of 5–6 weeks were purchased from Vital River Laboratories, Beijing, China. The female mice were housed in groups of four per cage and given food and water ad libitum. The housing room was under a 10L to 14D light/dark photoperiod (lights on at 9:00 am). The room temperature was maintained at 23 ± 2 °C. All the mice were acclimated to the new environment until 8 weeks of age.

### Superovulation and oocyte collection

The gonadotropins used in the current study were as follows: recombinant human follitropin for injection (r-hFSH, abbreviated as FSH below, Gonal-F ®, Merck Serono SA Aubonne Branch, Geneva, Switzerland), highly purified menotrophin for injection (hMG, Ferring GmbH, Witland, Kiel, Germany), and human chorionic gonadotropin (hCG, Huafu Biotechnology Ltd., Tianjin, China). The stock concentrations of FSH and hMG were 200 IU/mL and 50 IU/mL, respectively, while that of hCG was 5 IU/mL.

Naturally ovulated oocytes (*n* = 6) were taken from the cumulus-oocyte complexes (COCs) of estrous female mice.

Each experimental group of mice was intraperitoneally injected with different dosages of FSH (FSH 5 IU, *n* = 6; FSH 50 IU, *n* = 6; FSH 200 IU, *n* = 6) or hMG (hMG 5 IU, *n* = 6; hMG 50 IU, *n* = 6; hMG 200 IU, *n* = 7) for 48 h and primed with 0.1 mL 5 IU hCG. An hCG control group was also designed, and this group of mice was treated with only 0.1 mL 5 IU hCG (*n* = 6). Then, all these female mice were sacrificed approximately 14 h later by cervical dislocation to obtain ampullae, and COCs were collected from the oviduct ampullae.

All the COCs were digested by 0.2% hyaluronidase for a short time. Then, the denuded oocytes were treated with pronase to remove the zona pellucida and polar body. The MII oocytes from each mouse were treated separately, and the oocytes with the best morphology and status were selected for subsequent DNA methylation library preparation.

### DNA extraction and post-bisulfite adapter tagging (pBAT) library construction

The single-cell DNA methylation library of each oocyte was prepared similarly as previously described with minor alterations [54, 55]. Briefly, each individual oocyte was very carefully put into the bottom of a tube containing 4.5 μL lysate buffer with lambda DNA using a mouth pipette without forming any bubbles. After cell lysis for 3.5 h, they were subjected to bisulfite conversion using the MethyCode Bisulfite Conversion Kit (Thermo Scientific, cat. no. MECOV-50) according to the manufacturer’s instructions. The bisulfite-converted DNA was then preamplified using random nonamer primers with a truncated Illumina P5 adapter (5′- CTACACGACGCTCTTCCGATCTNNNNNN-3′) and 50 U/μL of Klenow polymerase (3 to 5 exo-, Enzymatics, cat. no. P7010-HC-L). The excess primers were removed using 2 μL of 20 U/μL exonuclease I (New England Biolabs, cat. no. M0293L) before DNA was purified using 0.9 × AMPure XP beads (Beckman Coulter, cat. no. A63881). The second strands were synthesized using 50 U/μL of Klenow polymerase (3 to 5 exo-, Enzymatics, cat. no. P7010-HC-L) with random nonamer primers containing a truncated P7 Illumina adapter (5′-AGACGTGTGCTCTTCCGATCTNNNNNN-3′) and purified using 0.9 × AMPure XP beads (Beckman Coulter, cat. no. A63881). The final library was amplified using KAPA HiFi HotStart ReadyMix (Kapabiosystems, cat. no. KK2602) with NEB primers (universal primer and index primer). Amplified libraries were purified twice with 0.9 × AMPure XP beads (Beckman Coulter, cat. no. A63881) and were quantified using Qubit ds HS dye and the Fragment Analyzer^TM^ Automated CE System (AATI). The final quality-ensured libraries were sequenced by Novogene on the Illumina X Ten sequencer with the 150 bp paired-end mode.

### Data processing and alignment

The analysis of single-cell DNA methylation data was carried out as previously described [56]. In brief, raw paired-end FASTQ reads were trimmed to remove the random primers, Illumina adapter sequences, and low-quality bases by Trim Galore in single-end mode (parameters: trim_galore --clip_R1 11 --quality 20 --stringency 3 --length 30). Trimmed reads were aligned to UCSC mm9 with Bismark in single-end-mode (parameters: bismark --bowtie2 --non_directional) [57]. The single-end mode mapped reads were merged, and PCR duplicates were removed for downstream analysis. The Bismark_methylation_extractor function in Bismark was used to perform methylation calls. Only samples with at least 10% mapping efficiency and more than 1 million CpG sites covered were included in the downstream analysis.

### Bisulfite conversion rate evaluation

Spiked lambda DNA was used to evaluate the bisulfite conversion rate of each sample. Specifically, the 48,502-bp lambda DNA genome was built as an extra reference with the bismark_genome_preparation function of Bismark. The trimmed reads were mapped to the built lambda genome, and CpG methylation was evaluated. The bisulfite conversion rate was calculated as the number of unmethylated Cs divided by the total number of Cs covered by the lambda genome sequencing.

### Oocyte methylation evaluation

The methylation level and variability of the annotated genomic regions were evaluated in the oocytes of each group. The coordinates of the annotated genomic regions (exon, intron, intergenic region, intragenic region, LINE, SINE, LTR, rRNA, tRNA) were downloaded from the UCSC genome browser. The oocyte-methylated and oocyte-unmethylated CGIs were downloaded from Kobayashi et al. [28]. The oocyte ICRs were also obtained from the paper of Kobayashi et al. [28]. The promoters were defined as the areas − 1500 bp and + 500 bp of any TSS, and only those promoters containing more than 8 CpGs were included in our analysis.

Due to the uneven distribution of CpG sites in the mouse genome, methylation evaluation of the mouse genome was performed in 100-CpG tiles. For the correlation analysis (Fig. 1f), the methylation level of the non-overlapping 100-CpG tiles across the whole genome was calculated by averaging the methylation levels of the CpG sites within the tiles, and the Pearson correlation coefficients for all of the samples were computed using the R command “cor” with “pairwise.complete.obs” as previously described [58].

### DMR analysis

Only the informative 100-CpG tiles were included in the DMR analysis. Specifically, in each pairwise comparison (one treatment group vs. one control group), only those tiles that covered at least 5 CpGs in at least 2 samples per group were included. First, DMRs were identified with the methylKit R package with difference = 20 and *q* value = 0.05 [59]. Second, to evaluate the false positive rate of the DMRs, we performed the DMR analysis with random combinations of the samples. The comparable DMR numbers indicate a high false positive rate even though the *q* value was filtered. Therefore, only those DMRs recurring in two or three comparisons in FSH [5 IU, 50 IU, 200 IU] vs. control (or hMG [5 IU, 50 IU, 200 IU] vs. control) were defined as DMRs. The identified DMRs were annotated by Homer with the default settings [60].

## Supplementary information

**Additional file 1: Figure S1.** Quality statistics of pBAT whole-genome DNA methylation sequencing data. **A** Mapping rate of each sample. **B** Bisulfite conversion rate of each sample. **C** Covered CpG number of each sample. **D** Histograms of the distribution of CpG methylation values for random samples of each group.

**Additional file 2: Figure S2** Analysis of CHG and CHH methylation levels. **A** Boxplot of whole-genome CHG methylation levels. **B** Boxplot of whole-genome CHH methylation levels.

**Additional file 3: Figure S3** Analysis of CG methylation levels in different genomic regions. **A** CG methylation levels of methylated CpG island (CGI) (metCGI), unmethylated CpG island (unmetCGI), promoter, exons, intron, intragenic region, intergenic region, LINE, SINE, LTR, rRNA, and tRNA. **B** CG methylation levels of maternal imprinting control regions (ICRs). Grey represents uncovered maternal ICRs.

**Additional file 4: Figure S4** Analysis of chromosome copy number. The chromosome copy number was not obviously influenced by superovulation with different dosages of FSH/hMG.

**Additional file 5: Figure S5** Relationship between the number of methylKit-identified DMRs and the corrected *p* value. **A** Number of DMRs vs. different corrected *p* values in real combinations and random combinations of all pairwise comparisons.

**Additional file 6: Supplementary Table S1.** Genes annotated in the hyper-/hypo-DMRs associated with superovulation with different dosages of FSH.

**Additional file 7: Figure S6** CpG distribution of the DMR containing *Gfod2* in the FSH group. **A** Integrative Genomic Viewer (IGV) screenshot showing the CpG distribution of the DMR at the *Gfod2* locus. Red: methylated. Blue: unmethylated.

**Additional file 8: Figure S7** Alterations in DNA methylation after superovulation with different dosages of FSH (vs. hCG control). **A** Heatmap of differentially methylated regions (DMRs) (100-CpG window size, corrected *p* value <0.05, difference > 20%) between the FSH and hCG control groups. Left panel, FSH 5 IU vs. hCG control. Middle panel, FSH 50 IU vs. hCG control. Right panel, FSH 200 IU vs. hCG control. **B** Integrative Genomic Viewer (IGV) screenshot of a 68 kb region showing methylation at the *Gfod2* locus, with one tile consistently hypermethylated in the FSH 5 IU, FSH 50 IU, and FSH 200 IU groups. Each vertical bar in the screenshot represents the methylation value (range, 0%-100%) of a non-overlapping 100-CpG tile. Genes are shown at the bottom of the screenshot. The treatment of each oocyte is shown on the right of the screenshot. **C** Boxplot of the methylation level of the consistently hypermethylated DMR shown in B.

**Additional file 9: Figure S8** Variability of methylation in different FSH groups. **A** Standard deviation of methylation level among biological replicates in controls and each FSH group. In each group, standard deviations of 10,000 100-CpG tiles per group were randomly selected for the boxplot. **B** Cumulative distribution curve of the standard deviation of the methylation level in each group. **C** Standard deviation of methylation level in high-, medium- and low-methylation CGIs in the oocytes of each group. **D** Distribution of the distance between the top 10,000 variable 100-CpG tiles and the nearest transcription start site (TSS). The nearest TSS of each tile was annotated with Homer2. More than 90% of the tiles in each group had a > 10K distance from the nearest TSS.

**Additional file 10: Supplementary Table S2.** Genes annotated in the hyper-/hypo-DMRs associated with superovulation with different dosages of hMG.

**Additional file 11: Figure S9** CpG distribution of the DMR containing *Gfod2* in the hMG group. **A** Integrative Genomic Viewer (IGV) screenshot showing the CpG distribution of the DMR at the *Gfod2* locus. Red: methylated. Blue: unmethylated.

**Additional file 12: Figure S10** Alterations in DNA methylation after superovulation with different dosages of hMG (vs. hCG control). **A** Heatmap of differentially methylated regions (DMRs) (100-CpG window size, corrected *p* value <0.05, difference > 20%) between the hMG and hCG control groups. Left panel, hMG 5 IU vs. hCG control. Middle panel, hMG 50 IU vs. hCG control. Right panel, hMG 200 IU vs. hCG control. **B** Integrative Genomic Viewer (IGV) screenshot of a 68 kb region showing methylation at the *Gfod2* locus, with one tile consistently hypermethylated in the hMG 5 IU, hMG 50 IU, and hMG 200 IU groups. Each vertical bar in the screenshot represents the methylation value (range, 0%-100%) of a non-overlapping 100-CpG tile. Genes are shown at the bottom of the screenshot. The treatment of each oocyte is shown on the right of the screenshot. **C** Boxplot of the methylation level of the consistently hypermethylated DMR shown in B.

**Additional file 13: Figure S11** Variability of methylation in different hMG groups. **A** Standard deviation of methylation level among biological replicates in controls and each hMG group. In each group, standard deviations of 10,000 100-CpG tiles per group were randomly selected for the boxplot. **B** Cumulative distribution curve of the standard deviation of the methylation level in each group. **C** Standard deviation of methylation level in high-, medium- and low-methylation CGIs in the oocytes of each group. **D** Distribution of the distance between the top 10,000 variable 100-CpG tiles and the nearest transcription start site (TSS). The nearest TSS of each tile was annotated with Homer2. More than 90% of the tiles in each group had a > 10K distance from the nearest TSS.

**Additional file 14: Supplementary Table S3.** Overlapped genes annotated in the DMRs between the same dosage of FSH and hMG treatment.

## Data Availability

The data used in the current study are as follows: GSE136730.

## Abbreviations

ARTs: Assisted reproductive technologies
BWS: Beckwith-Wiedemann syndrome
SRS: Silver-Russell syndrome
AS: Angelman syndrome
PWS: Prader-Willi syndromes
GV: Germinal vesicle
DNMT3A: DNA methyltransferases 3A
DNMT3L: DNA methyltransferases 3 L
CGIs: CpG islands
gDMRs: Germline differentially methylated regions
FSH: Follicle-stimulating hormone
hMG: Human menopausal gonadotropin
hCG: Human chorionic gonadotropin
MII: Metaphase II
TSS: Transcription start site
TTS: Transcript terminate site
metCGIs: Methylated CpG islands
unmetCGIs: Unmethylated CpG islands
ICRs: Imprinting control regions
IGV: Integrative Genomic Viewer
Gfod2: Glucose-fructose oxidoreductase domain containing 2
Celf4: CUGBP, ELAV-like family member 4
KRAB-ZFP: Kruppel-associated box-zinc finger protein
Sox17: SRY-related HMG box gene 17
Shroom3: Shroom family member 3
Phactr4: Phosphatase and actin regulator 4
COCs: Cumulus-oocyte complexes
pBAT: Post-bisulfite adapter tagging
AATI: Fragment Analyzer^TM^ Automated CE System
