# Supplementary figures and images for: Single-cell DNA methylation sequencing reveals epigenetic alterations in mouse oocytes superovulated with different dosages of gonadotropins
Source: Clin Epigenetics. 2020 Jun 1;12:75. doi: 10.1186/s13148-020-00866-w (PMC7268365; doi:10.1186/s13148-020-00866-w)

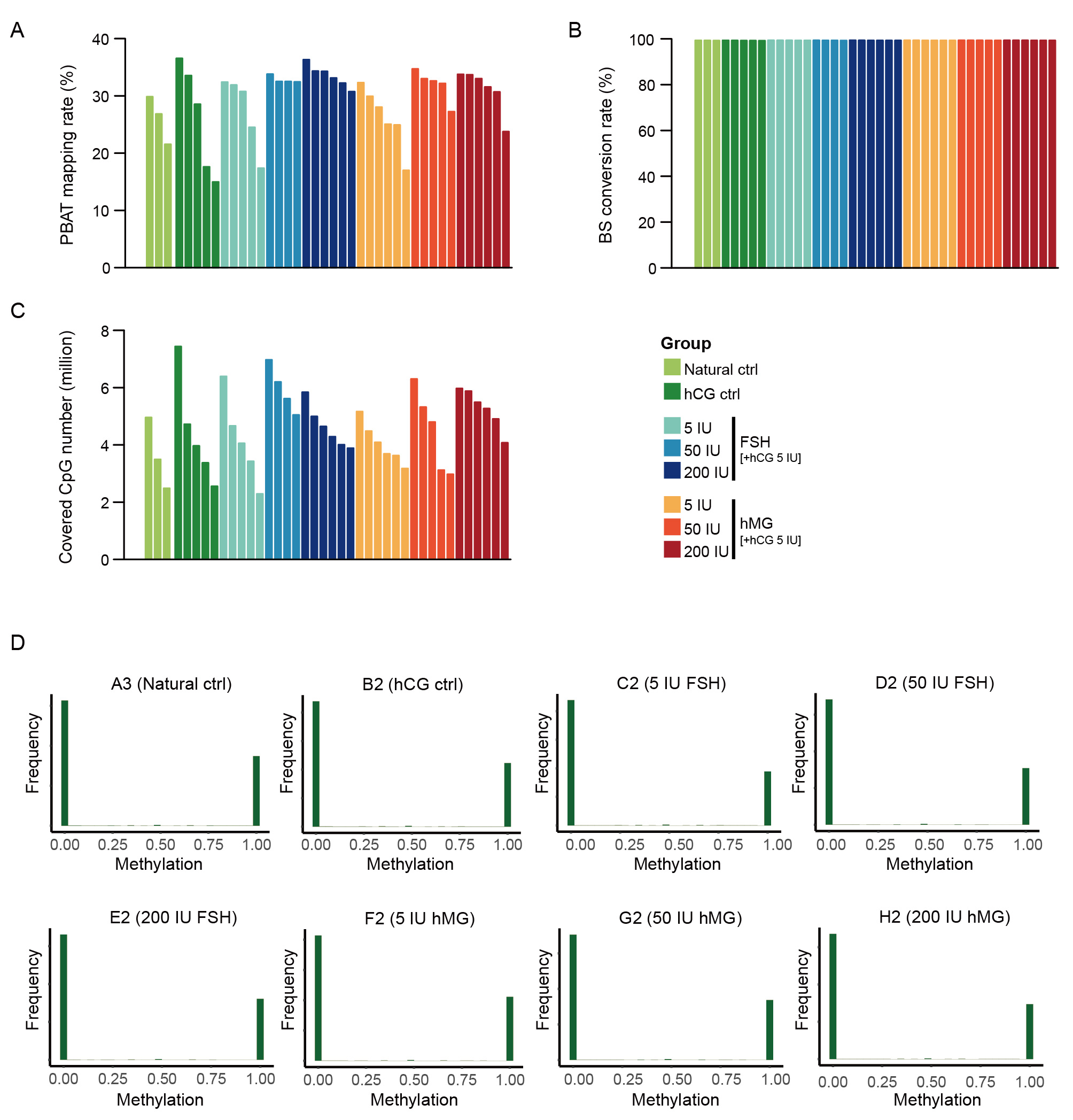

Supplement: Supplementary file 1 — Additional file 1: Figure S1. Quality statistics of pBAT whole-genome DNA methylation sequencing data. A Mapping rate of each sample. B Bisulfite conversion rate of each sample. C Covered CpG number of each sample. D Histograms of the distribution of CpG methylation values for random samples of each group. [file 13148_2020_866_MOESM1_ESM.jpg]

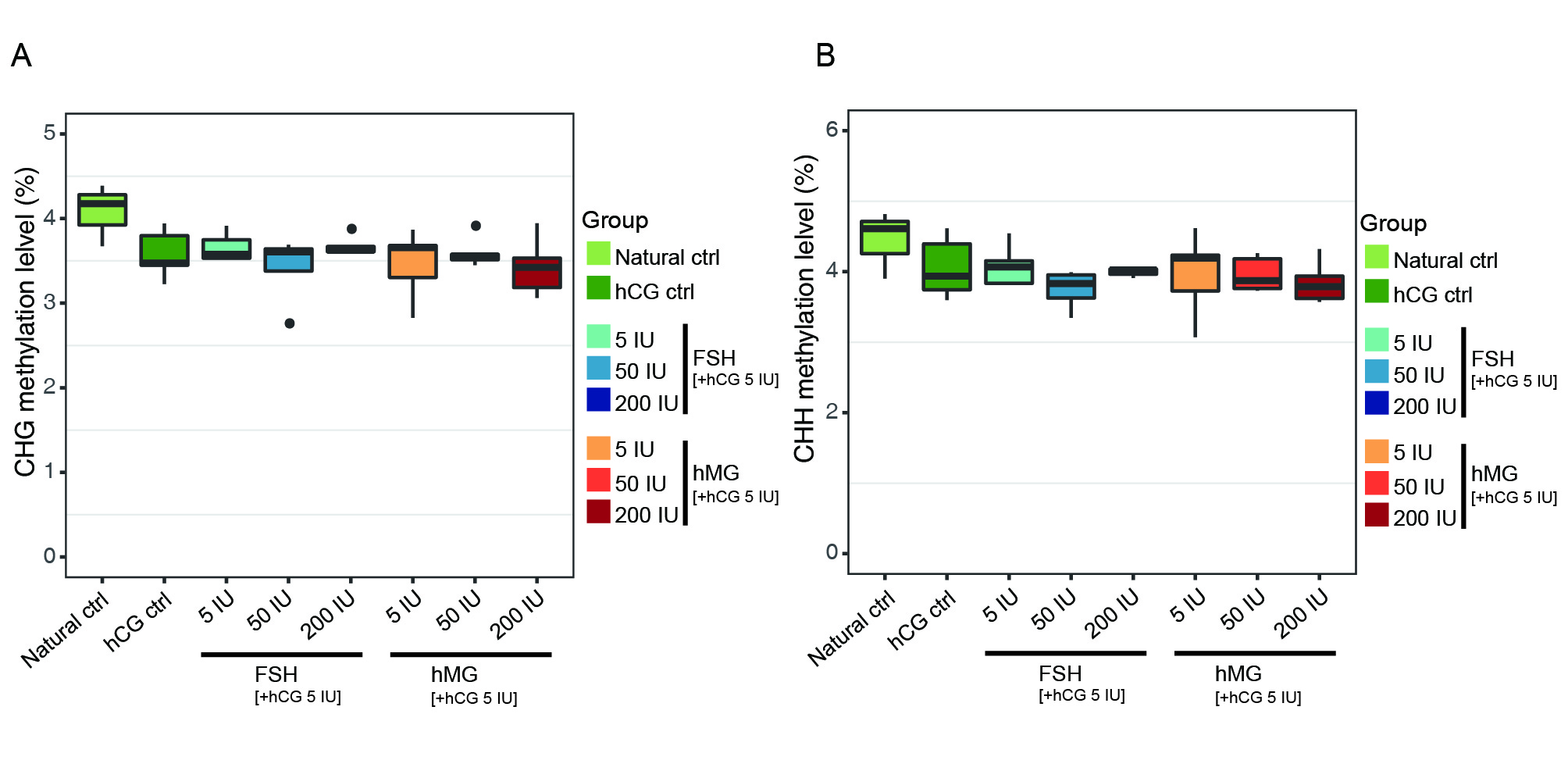

Supplement: Supplementary file 2 — Additional file 2: Figure S2 Analysis of CHG and CHH methylation levels. A Boxplot of whole-genome CHG methylation levels. B Boxplot of whole-genome CHH methylation levels. [file 13148_2020_866_MOESM2_ESM.jpg]

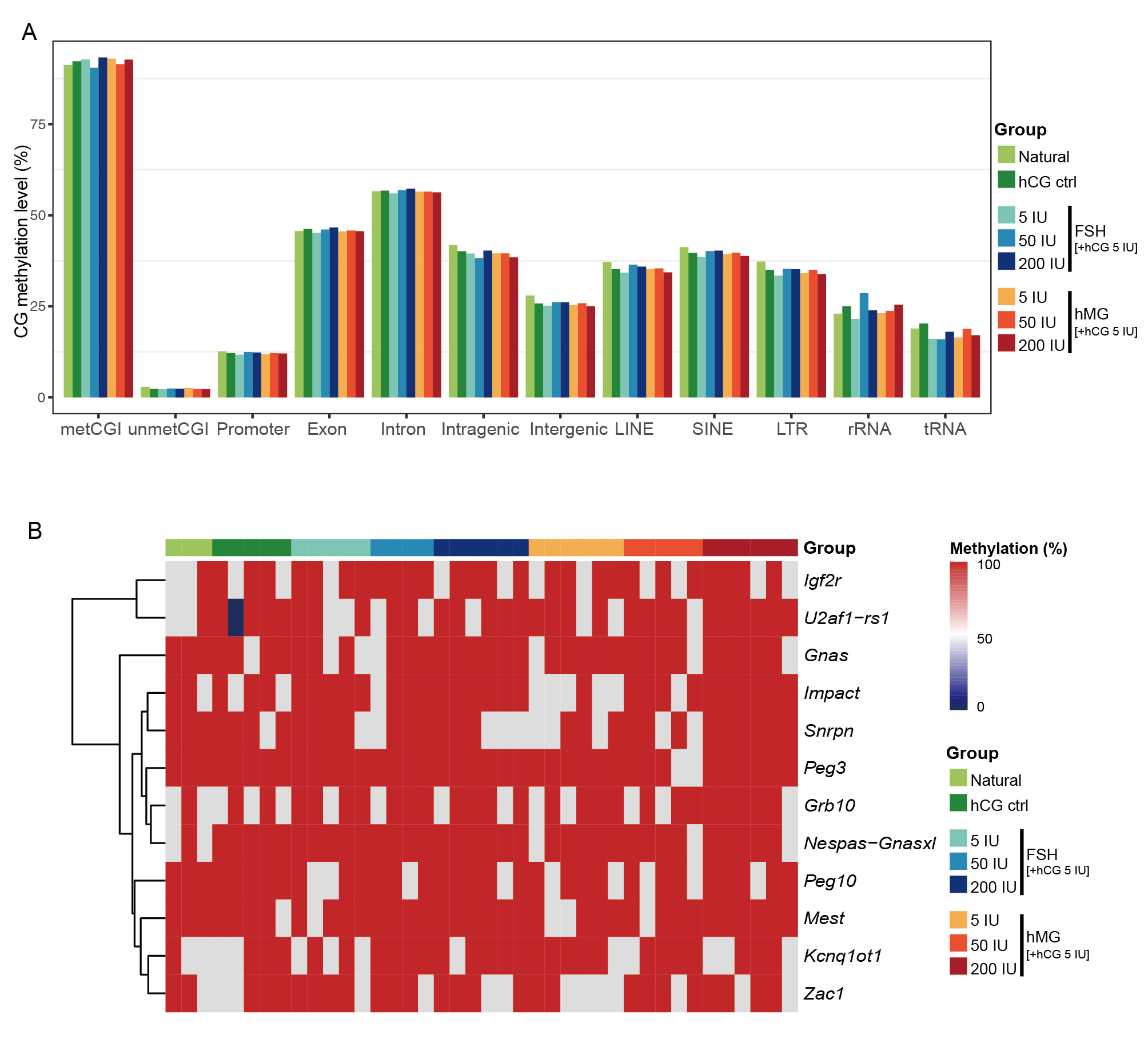

Supplement: Supplementary file 3 — Additional file 3: Figure S3 Analysis of CG methylation levels in different genomic regions. A CG methylation levels of methylated CpG island (CGI) (metCGI), unmethylated CpG island (unmetCGI), promoter, exons, intron, intragenic region, intergenic region, LINE, SINE, LTR, rRNA, and tRNA. B CG methylation levels of maternal imprinting control regions (ICRs). Grey represents uncovered maternal ICRs. [file 13148_2020_866_MOESM3_ESM.jpg]

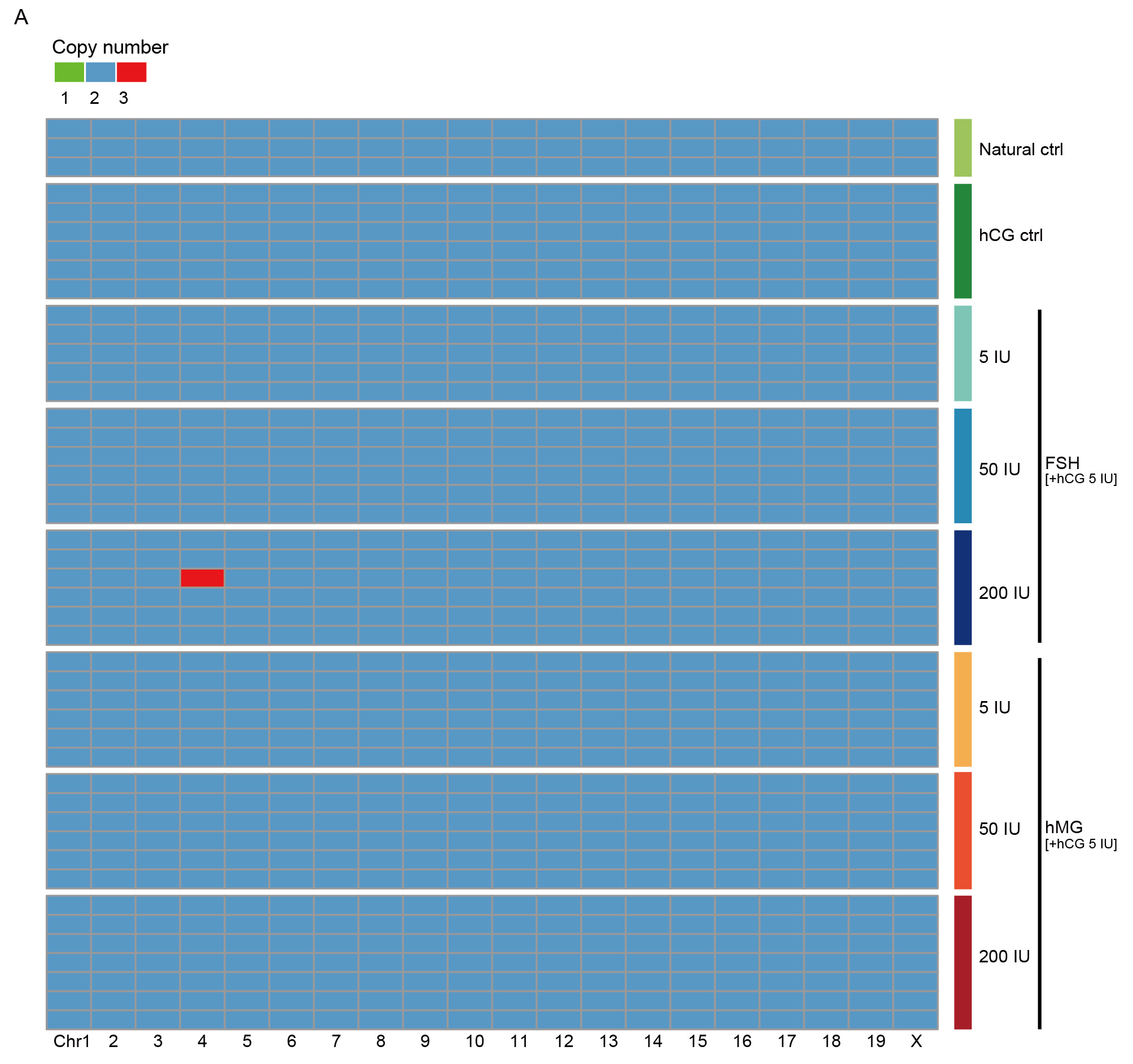

Supplement: Supplementary file 4 — Additional file 4: Figure S4 Analysis of chromosome copy number. The chromosome copy number was not obviously influenced by superovulation with different dosages of FSH/hMG. [file 13148_2020_866_MOESM4_ESM.jpg]

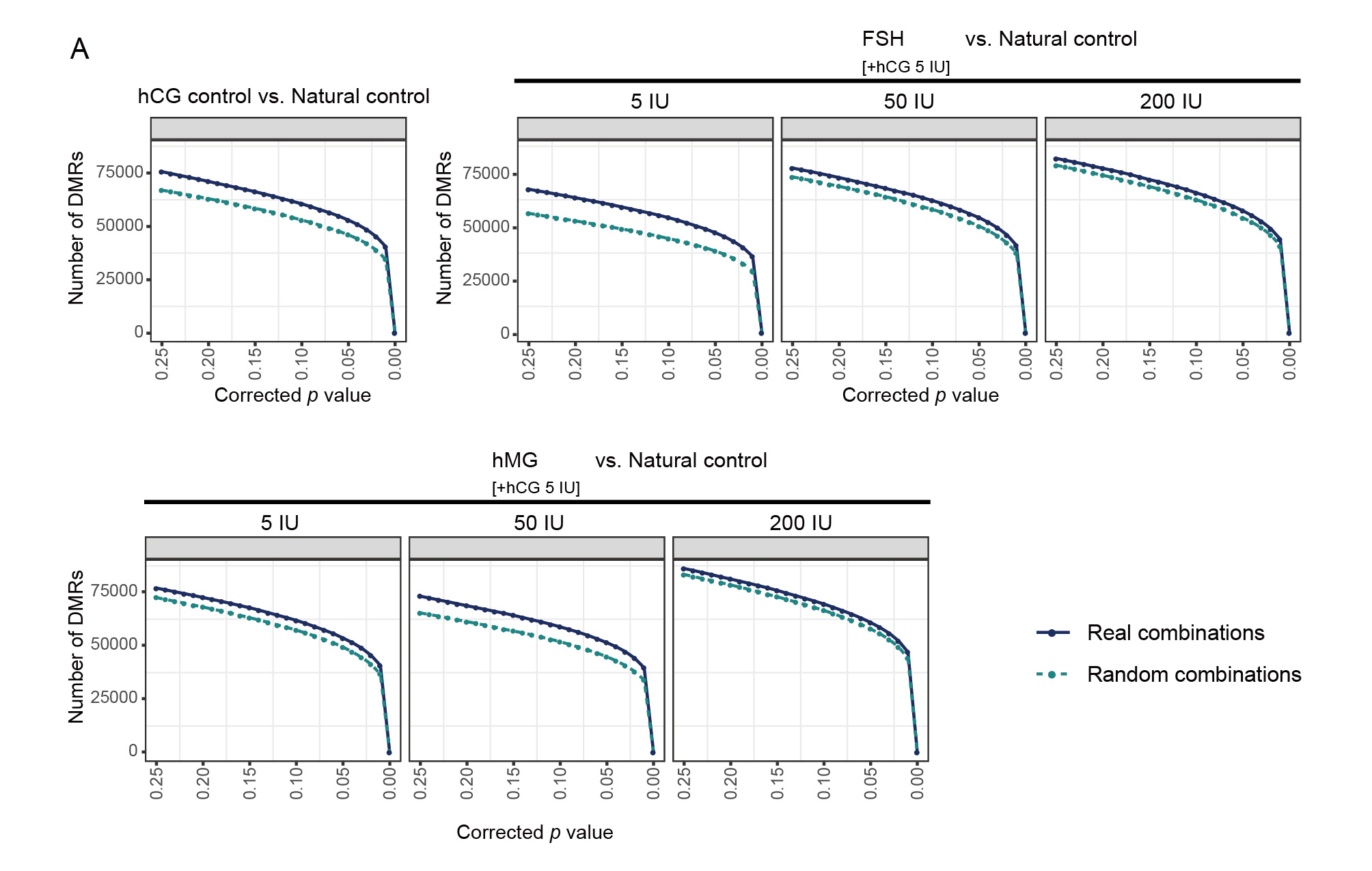

Supplement: Supplementary file 5 — Additional file 5: Figure S5 Relationship between the number of methylKit-identified DMRs and the corrected p value. A Number of DMRs vs. different corrected p values in real combinations and random combinations of all pairwise comparisons. [file 13148_2020_866_MOESM5_ESM.jpg]

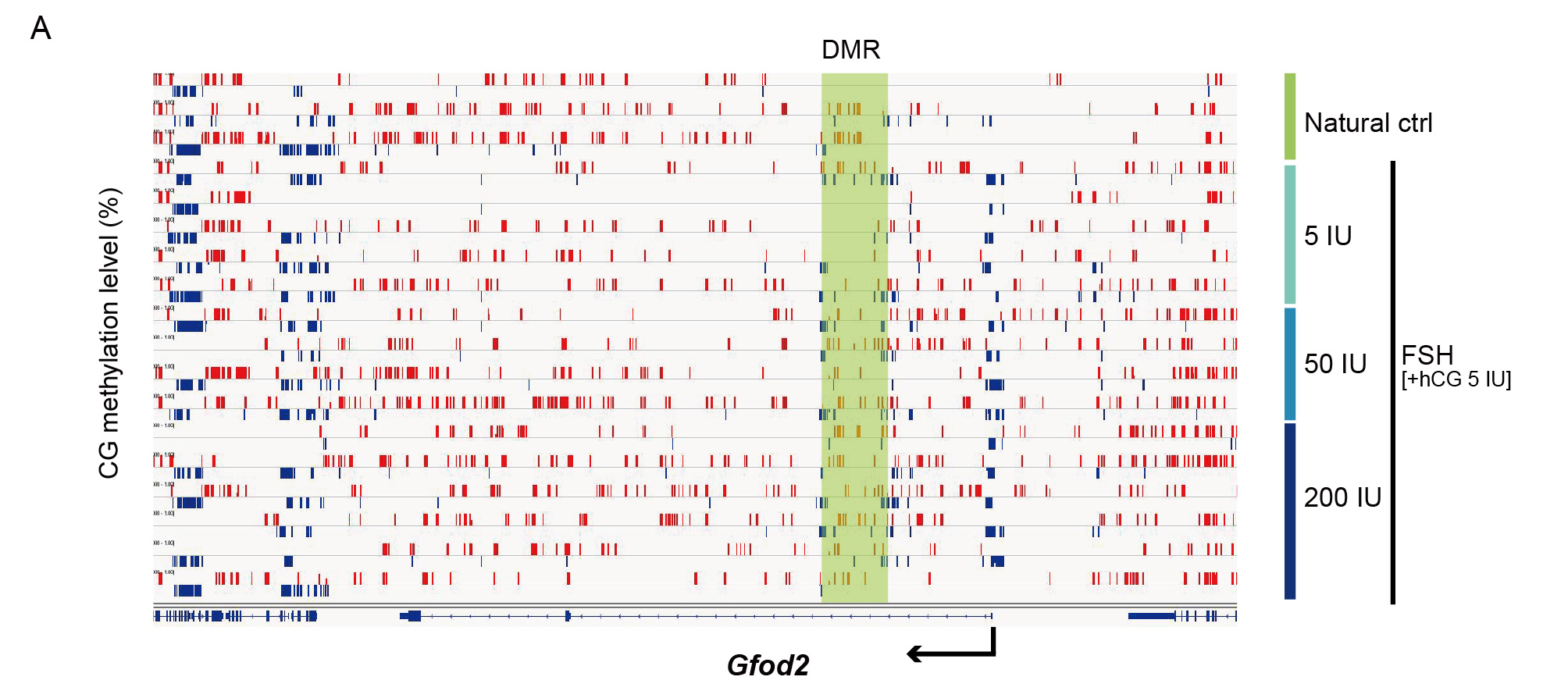

Supplement: Supplementary file 7 — Additional file 7: Figure S6 CpG distribution of the DMR containing Gfod2 in the FSH group. A Integrative Genomic Viewer (IGV) screenshot showing the CpG distribution of the DMR at the Gfod2 locus. Red: methylated. Blue: unmethylated. [file 13148_2020_866_MOESM7_ESM.jpg]

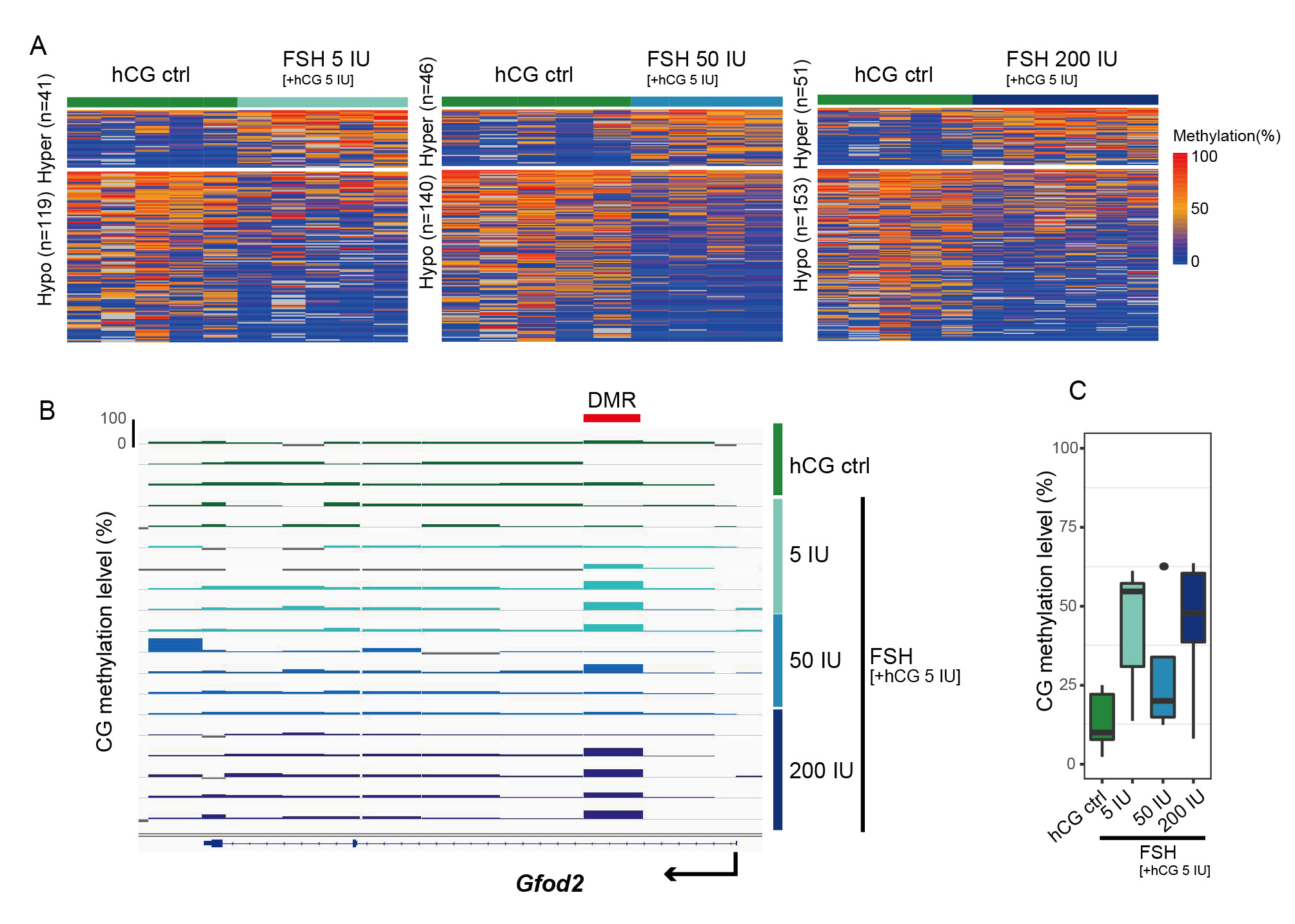

Supplement: Supplementary file 8 — Additional file 8: Figure S7 Alterations in DNA methylation after superovulation with different dosages of FSH (vs. hCG control). A Heatmap of differentially methylated regions (DMRs) (100-CpG window size, corrected p value <0.05, difference > 20%) between the FSH and hCG control groups. Left panel, FSH 5 IU vs. hCG control. Middle panel, FSH 50 IU vs. hCG control. Right panel, FSH 200 IU vs. hCG control. B Integrative Genomic Viewer (IGV) screenshot of a 68 kb region showing methylation at the Gfod2 locus, with one tile consistently hypermethylated in the FSH 5 IU, FSH 50 IU, and FSH 200 IU groups. Each vertical bar in the screenshot represents the methylation value (range, 0%-100%) of a non-overlapping 100-CpG tile. Genes are shown at the bottom of the screenshot. The treatment of each oocyte is shown on the right of the screenshot. C Boxplot of the methylation level of the consistently hypermethylated DMR shown in B. [file 13148_2020_866_MOESM8_ESM.jpg]

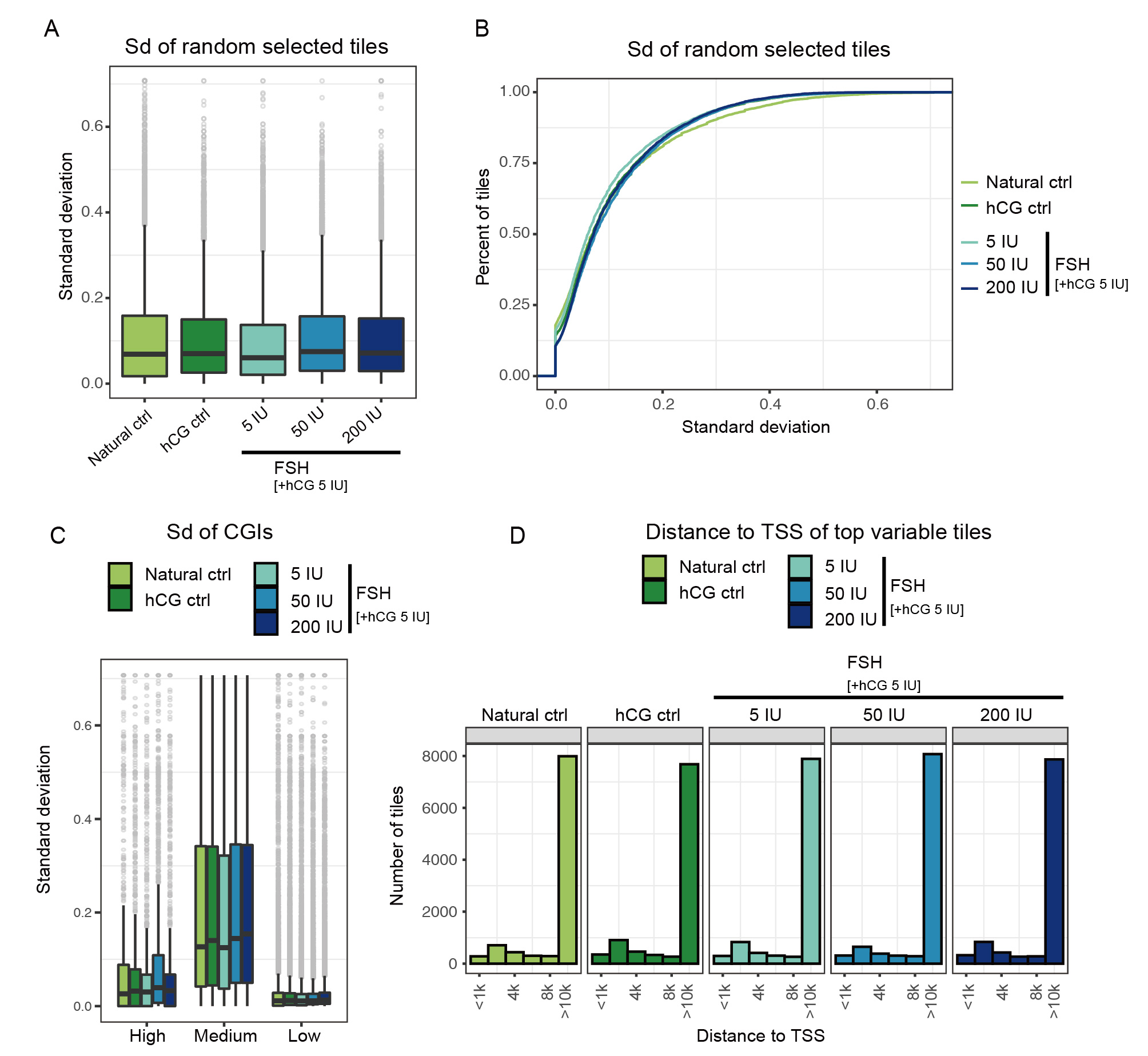

Supplement: Supplementary file 9 — Additional file 9: Figure S8 Variability of methylation in different FSH groups. A Standard deviation of methylation level among biological replicates in controls and each FSH group. In each group, standard deviations of 10,000 100-CpG tiles per group were randomly selected for the boxplot. B Cumulative distribution curve of the standard deviation of the methylation level in each group. C Standard deviation of methylation level in high-, medium- and low-methylation CGIs in the oocytes of each group. D Distribution of the distance between the top 10,000 variable 100-CpG tiles and the nearest transcription start site (TSS). The nearest TSS of each tile was annotated with Homer2. More than 90% of the tiles in each group had a > 10K distance from the nearest TSS. [file 13148_2020_866_MOESM9_ESM.jpg]

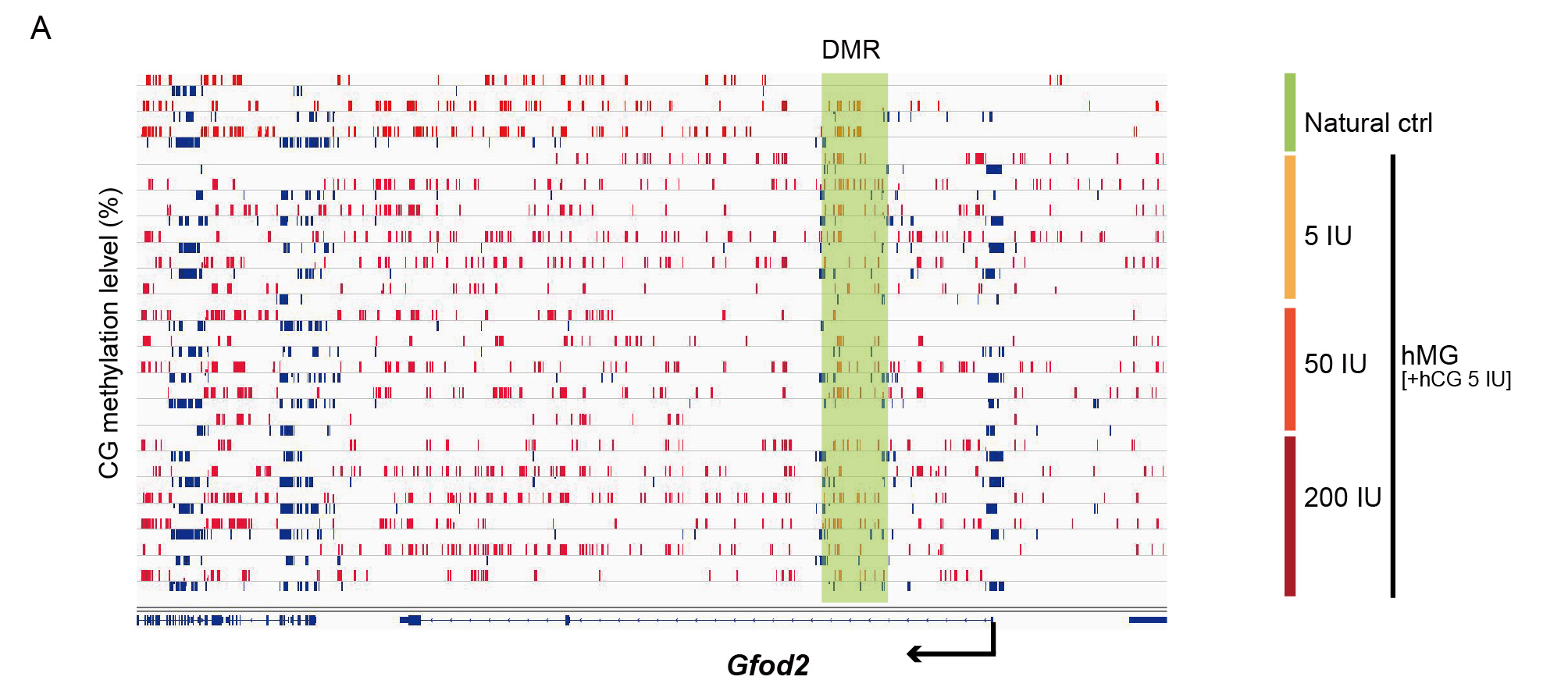

Supplement: Supplementary file 11 — Additional file 11: Figure S9 CpG distribution of the DMR containing Gfod2 in the hMG group. A Integrative Genomic Viewer (IGV) screenshot showing the CpG distribution of the DMR at the Gfod2 locus. Red: methylated. Blue: unmethylated. [file 13148_2020_866_MOESM11_ESM.jpg]

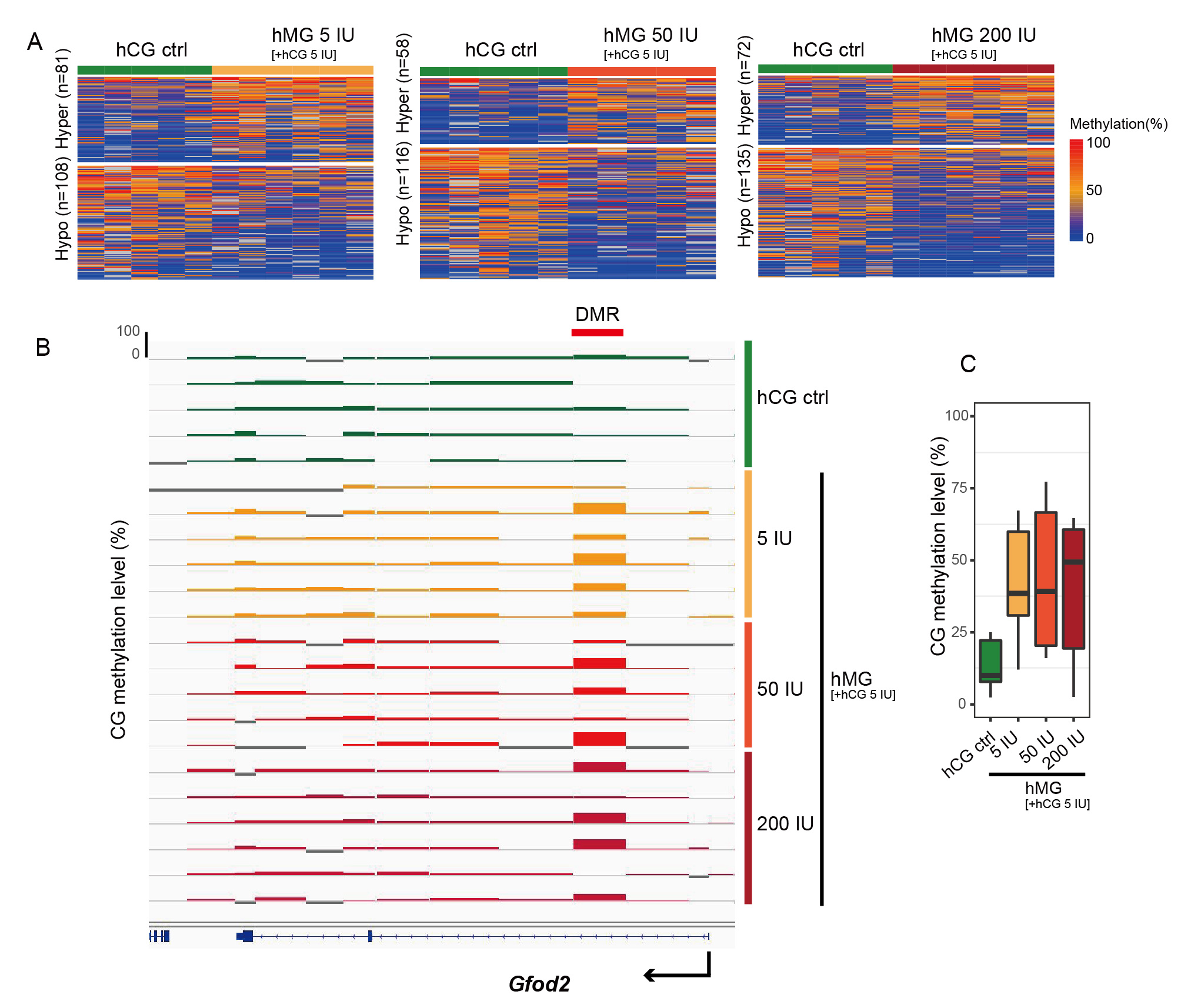

Supplement: Supplementary file 12 — Additional file 12: Figure S10 Alterations in DNA methylation after superovulation with different dosages of hMG (vs. hCG control). A Heatmap of differentially methylated regions (DMRs) (100-CpG window size, corrected p value <0.05, difference > 20%) between the hMG and hCG control groups. Left panel, hMG 5 IU vs. hCG control. Middle panel, hMG 50 IU vs. hCG control. Right panel, hMG 200 IU vs. hCG control. B Integrative Genomic Viewer (IGV) screenshot of a 68 kb region showing methylation at the Gfod2 locus, with one tile consistently hypermethylated in the hMG 5 IU, hMG 50 IU, and hMG 200 IU groups. Each vertical bar in the screenshot represents the methylation value (range, 0%-100%) of a non-overlapping 100-CpG tile. Genes are shown at the bottom of the screenshot. The treatment of each oocyte is shown on the right of the screenshot. C Boxplot of the methylation level of the consistently hypermethylated DMR shown in B. [file 13148_2020_866_MOESM12_ESM.jpg]

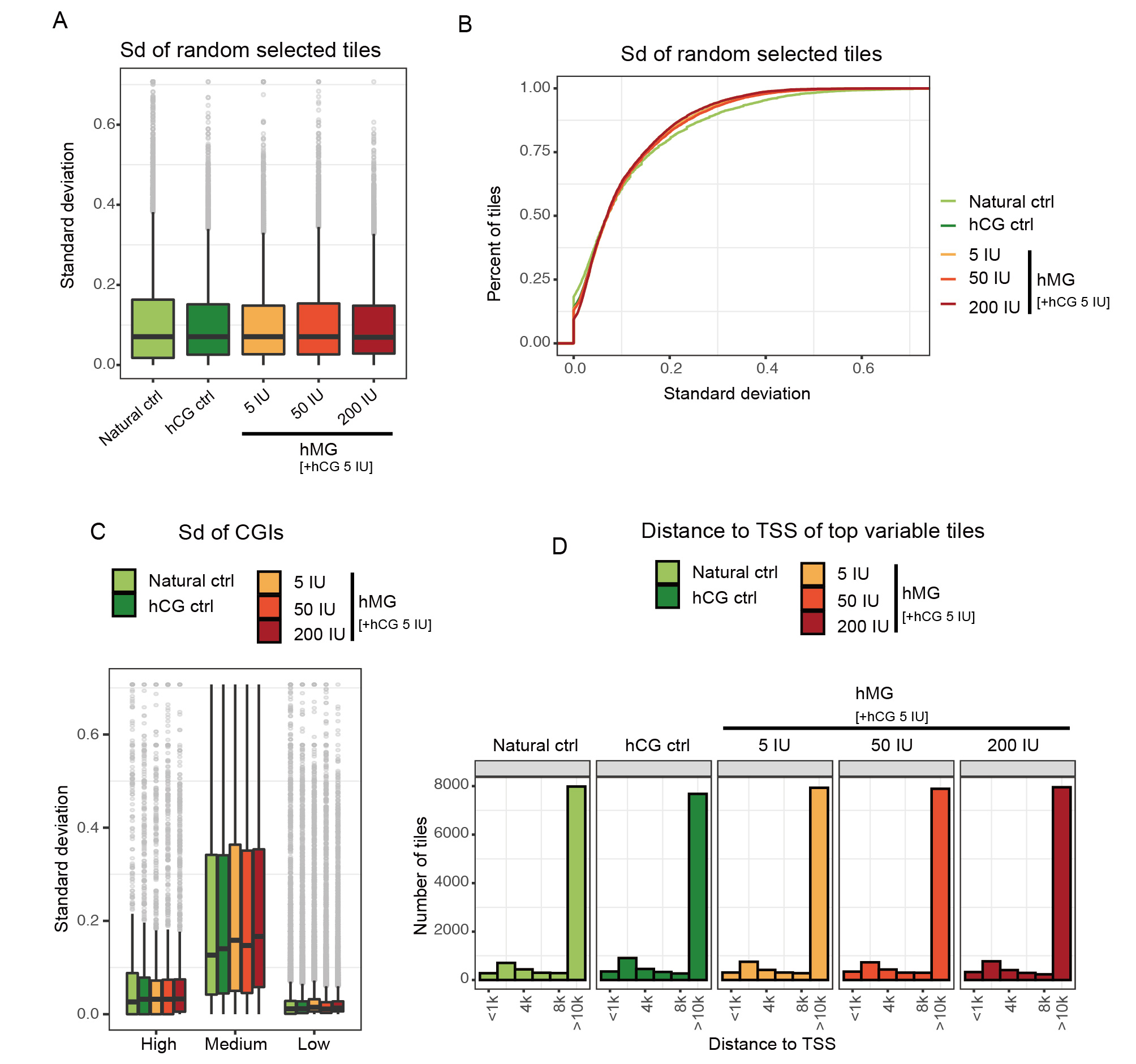

Supplement: Supplementary file 13 — Additional file 13: Figure S11 Variability of methylation in different hMG groups. A Standard deviation of methylation level among biological replicates in controls and each hMG group. In each group, standard deviations of 10,000 100-CpG tiles per group were randomly selected for the boxplot. B Cumulative distribution curve of the standard deviation of the methylation level in each group. C Standard deviation of methylation level in high-, medium- and low-methylation CGIs in the oocytes of each group. D Distribution of the distance between the top 10,000 variable 100-CpG tiles and the nearest transcription start site (TSS). The nearest TSS of each tile was annotated with Homer2. More than 90% of the tiles in each group had a > 10K distance from the nearest TSS. [file 13148_2020_866_MOESM13_ESM.jpg]
